# Graph generative and adversarial strategy-enhanced node feature learning and self-calibrated pairwise attribute encoding for prediction of drug-related side effects

**DOI:** 10.3389/fphar.2023.1257842

**Published:** 2023-09-04

**Authors:** Ping Xuan, Kai Xu, Hui Cui, Toshiya Nakaguchi, Tiangang Zhang

**Affiliations:** ^1^ Department of Computer Science, School of Engineering, Shantou University, Shantou, China; ^2^ School of Computer Science and Technology, Heilongjiang University, Harbin, China; ^3^ Department of Computer Science and Information Technology, La Trobe University, Melbourne, VI, Australia; ^4^ Center for Frontier Medical Engineering, Chiba University, Chiba, Japan; ^5^ School of Mathematical Science, Heilongjiang University, Harbin, China

**Keywords:** graph generative and adversarial strategy, topologies and attributes from heterogeneous graphs, graph convolutional autoencoder, self-calibrated pairwise attributes, representation-level attention

## Abstract

**Background:** Inferring drug-related side effects is beneficial for reducing drug development cost and time. Current computational prediction methods have concentrated on graph reasoning over heterogeneous graphs comprising the drug and side effect nodes. However, the various topologies and node attributes within multiple drug–side effect heterogeneous graphs have not been completely exploited.

**Methods:** We proposed a new drug-side effect association prediction method, GGSC, to deeply integrate the diverse topologies and attributes from multiple heterogeneous graphs and the self-calibration attributes of each drug-side effect node pair. First, we created two heterogeneous graphs comprising the drug and side effect nodes and their related similarity and association connections. Since each heterogeneous graph has its specific topology and node attributes, a node feature learning strategy was designed and the learning for each graph was enhanced from a graph generative and adversarial perspective. We constructed a generator based on a graph convolutional autoencoder to encode the topological structure and node attributes from the whole heterogeneous graph and then generate the node features embedding the graph topology. A discriminator based on multilayer perceptron was designed to distinguish the generated topological features from the original ones. We also designed representation-level attention to discriminate the contributions of topological representations from multiple heterogeneous graphs and adaptively fused them. Finally, we constructed a self-calibration module based on convolutional neural networks to guide pairwise attribute learning through the features of the small latent space.

**Results:** The comparison experiment results showed that GGSC had higher prediction performance than several state-of-the-art prediction methods. The ablation experiments demonstrated the effectiveness of topological enhancement learning, representation-level attention, and self-calibrated pairwise attribute learning. In addition, case studies over five drugs demonstrated GGSC’s ability in discovering the potential drug-related side effect candidates.

**Conclusion:** We proposed a drug-side effect association prediction method, and the method is beneficial for screening the reliable association candidates for the biologists to discover the actual associations.

## 1 Introduction

Drug-related side effects are harmful outcomes that go beyond the therapeutic expectations of a drug’s application, which can result in its failure during clinical studies (Ding et al., 2018; Cakir et al., 2021; Zhang et al., 2021). Therefore, recognizing drugs’ adverse effects might help to minimize drug development cost and time (Jiang et al., 2018; Sachdev and Gupta, 2020). Computational prediction methods have proven helpful in selecting suitable drug-related side effect candidates for biological testing.

Existing studies can be grouped into three main categories. The first category uses drug-related biological data to forecast potential side effects. Francesco et al. and Wishart et al. exploited the similarity of gene expression profiles of multiple drug-treated cell lines to predict unexpected adverse drug reactions (Iorio et al., 2010; Li et al., 2016). However, these two methods are limited by unknown molecular differences (Wishart et al., 2008). Therefore, applying such methods on a large scale to predict reliable drug-related side effect candidates is difficult (Ma et al., 2003; Pauwels et al., 2011; Sawada et al., 2015).

The second category uses machine learning-based models to predict associations between drug use and adverse effects. Pauwels et al. used four machine learning methods to build prediction models: support vector machine, k-nearest neighbor (KNN), ordinary canonical correlation analysis, and sparse canonical correlation analysis (Bresso et al., 2013). A feature-derived graph regularization matrix decomposition method was proposed to predict side effects not found based on accessible drug attributes and known drug–side effect connections in medications at present (Dimitri and Lió, 2017). Decision trees and inductive logic methods were introduced by Bresso et al. (Uner et al., 2019). Zhang et al. inferred potential side effect associations for drugs using a feature selection-based multi-label KNN method (Xu et al., 2022). In addition, Cakir et al. and Dimitri et al. used random forest and Bayesian algorithms to predict drugs’ potential side effects, respectively (Seo et al., 2020; Joshi et al., 2022). However, these methods are shallow predictive models that cannot effectively learn deeper correlations between nodes.

The category uses deep learning to combine more detailed information between nodes and enhance model forecast performance. Uner et al. developed four prediction models using a multilayer perceptron (MLP), multi-modal neural networks, multi-task neural networks, and simplified molecular input line entry system convolutional neural networks, respectively (Lee et al., 2017; Zheng et al., 2019). Some studies have combined similarity data between drugs and their side effects and estimated the frequency of pharmacological side effects using deep neural (Yang et al., 2009) and graph attention neural (Luo et al., 2011; Liu et al., 2012; Mizutani et al., 2012) networks. However, these methods disregard the value of heterogeneous graphs comprising several associations between drugs and side effects when attempting to anticipate potentially important pharmacological side effects (Zhang et al., 2015; Ding et al., 2019). They proposed a graph convolutional neural network combining graph and node embedding to improve model prediction performance. In addition, adverse drug reactions have also been predicted using deep neural networks based on knowledge graph embedding (Zhang et al., 2018; Zhao et al., 2018; Hu et al., 2019). However, this approach ignores the extraction of enhanced topological representations through adversarial learning and the learning of attributes of node pairs after self-calibration.

In this study, we present a new drug-related side effect prediction method, GGSC, which learns the topological features of drug and side effect nodes enhanced by the generative and adversarial strategy and integrates the self-calibration attributes of each drug–side effect node pair. The contributions of our prediction method are listed as follows.

First, for each heterogeneous graph, a generative adversarial-based strategy is designed to learn the topological representations of the drug and side effect nodes. In this way, these representations are learned and enhanced from the whole graph perspective.

Second, the generator comprises a graph convolutional encoder and decoder to generate a false topological embedding of all the drug and side effect nodes. The encoder based on graph convolutional neural network encodes the topological structure and node attributes of each heterogeneous graph.

Third, the decoder generates the false topological embedding according to the encoded feature map. The discriminator contains multilayer perceptron to determine whether the topological embedding is the original feature one or the generated false one. The encoded topological features and node attributes of the drug and side effect nodes are enhanced by the generative and adversarial strategy.

Finally, a self-calibrating convolutional neural network (SCC)-based module is constructed to learn the attributes of each drug–side effect node pair from multiple heterogeneous graphs. More global information is obtained through greater receptive field in a small latent feature space, and it is utilized to guide the pairwise feature learning in an original feature space.

## 2 Materials and methods

**FIGURE 1:**
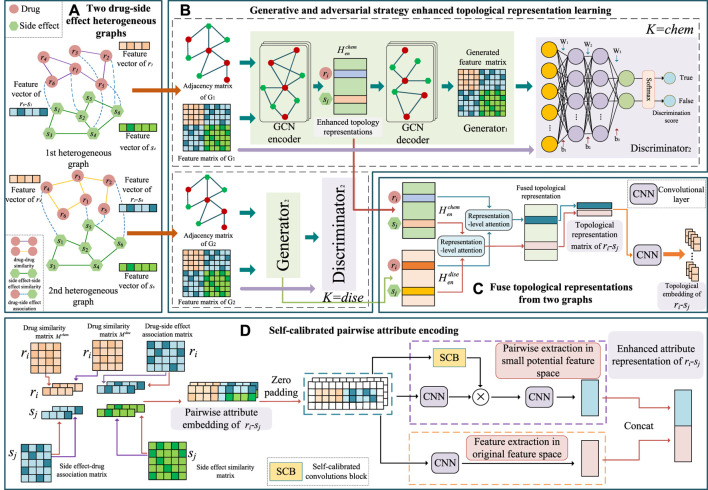
Framework of the proposed GGSC model. **(A)** Two drug–side effect heterogeneous graphs constructed based on two kinds of drug similarities. **(B)** Enhanced topological representation learning via generative and adversarial networks based on graph convolutional autoencoders. **(C)** Topological fusion based on representation-level attention. **(D)** Pairwise attribute representation learning by self-calibrated convolutional neural networks.

**FIGURE 2:**
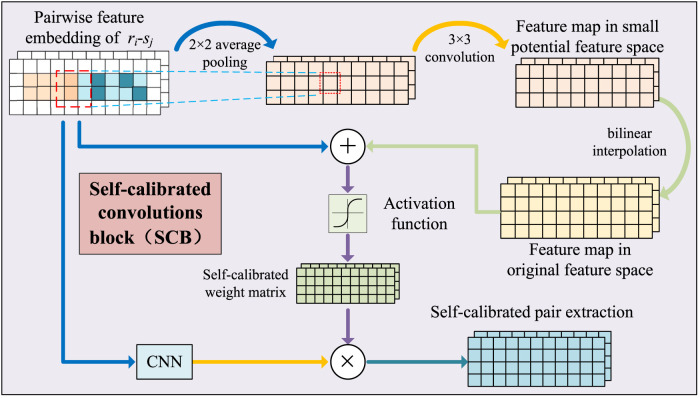
Illustration of pairwise attribute learning based on self-calibrated convolutional neural networks.

Our primary goal is to predict a drug’s probable relevant side effects. We built a GGSC model comprising an SCC and a generative adversarial network (GAN) with a representational-level attention mechanism based on information from many sources about drugs and their adverse effects. The model comprises two branches. To thoroughly understand the topological representation of nodes, we first built two distinct bilayer heterogeneous networks based on two drug similarities, side effect similarities, and drug–side effect associations (Figure 1A). In the first branch, we learn the topological representation of network-level enhancements in the two heterogeneous graphs based on GANs. The learned topological representation is then integrated using a novel attention method, and drug–side effect node pairs are extracted to obtain association prediction scores via convolution and fully connected layers. We used an SCC to encode the specifics and characteristics of the other branch’s self-calibrated drug–side effect node pairs (Figure 2). The prediction scores of the last two branches were combined by weighting to obtain the final association score, which reflects the likelihood of the drug having the corresponding side effects.

### 2.1 Dataset

Datasets were obtained from the work of Galeano et al. (2020), Guo et al. (2020), and Zhao et al. (2021), originally collected from the side effect resource (SIDER) and comparative toxicogenomics databases. They include drug similarities, drug–side effect associations, and drug–disease relationships. We examined 4,192 side effects from 708 drugs, representing 80,164 known pairs of associations in the SIDER database. We extracted 199,214 drug–disease pairs from the comparative toxicogenomics database, representing 708 drugs and 5,603 diseases. Drug similarity was based on chemical substructure calculations.

### 2.2 Matrix expressions of multi-source data about the drugs and side effects

#### 2.2.1 Drug–side effect heterogeneous graph

Two separate drug–side effect heterogeneous graphs were created for two drugs with similar chemical properties based on chemical substructures and drug-related disorders. The two graphs are denoted as *G* ^*chem*^ = (*V* ^*chem*^, *E* ^*chem*^) and *G* ^*dise*^ = (*V* ^*dise*^, *E* ^*dise*^), where the set of nodes *V* = {*V* ^*m*^ ∪ *V* ^*s*^} comprises the set of drug nodes *V* ^*m*^ and the set of side effect nodes *V* ^*s*^. The edge set *E* comprises the edges between nodes, with the edges between nodes *v*_*i*_ and *v*_*j*_ denoted by *e*_*ij*_ ∈ *E*. The heterogeneous graphs *G* ^*chem*^ and *G* ^*dise*^ contain three edge types: drug–drug similarity linkage edges, side effect–side effect similarity linkage edges, and drug–side effect association edges.

#### 2.2.2 Expressions of the similarities and associations among the drugs and side effects

##### Drug similarity matrix

Based on the drug’s chemical makeup and associated disorders, we obtained two drug similarity matrices, defined as follows,

$$M^{k}=\left\{\begin{array}{c} M^{\mathrm{chem}}={\left(M^{\mathrm{chem}}\right)}_{ij}\in R^{N_{r}\times N_{r}},\;\\ M^{\mathrm{dise}}={\left(M^{\mathrm{dise}}\right)}_{ij}\in R^{N_{r}\times N_{r}},\; \end{array}\right.$$ (1)

where *N*_*r*_ is the number of drugs and *M* ^*k*^(*k* = *chem*, *dise*) is the degree of similarity determined based on the drug’s chemical makeup and the disease it treats.

When two drugs *r*_*i*_ and *r*_*j*_ have more common chemical substructures, their functions are usually more similar. Based on this biological premise, the previous methods (Liang et al., 2017; Zhao et al., 2022) calculated the drug similarities by the cosine similarity measure on their chemical substructures. When calculating *M* ^*dise*^, two drugs share more associated diseases and have a higher similarity. Using Wang et al.’s method, taking drugs *r*_*i*_ and *r*_*j*_ as an example, we first obtain the disease set $D_{ri}=\left\{d_{i1},d_{i2}\cdots d_{in}\right\}$ associated with *r*_*i*_ and the disease set $D_{rj}=\left\{d_{j1},d_{j2}\cdots d_{jm}\right\}$ associated with *r*_*j*_. We then take the similarity between *D*_*ri*_ and *D*_*rj*_ as the similarity between drugs *r*_*i*_ and *r*_*i*_.

The matrix *S* depicts the side effect similarities.

$$S={\left(S\right)}_{ij}\in R^{N_{s}\times N_{s}},$$ (2)

where *N*_*s*_ represents the number of nodes with side effects. Side effects *s*_*i*_ and *s*_*j*_ are more likely to be similar when they share more associated drugs. Therefore, using the technique of Wang et al., first of all, we obtained the drug sets *M*_*si*_ and *M*_*sj*_ associated with side effects *s*_*i*_ and *s*_*j*_. Then, we calculated the similarity between the drug sets *M*_*si*_ and *M*_*sj*_, and the outcome served as a measure of how closely side effects *s*_*i*_ and *s*_*j*_ are related. The side effect similarity matrix was then obtained. (*S*)_*ij*_ indicates the degree of similarity between *s*_*i*_ and *s*_*j*_, varying from 0 to 1; the higher the value, the higher the corresponding similarity.

The matrix *O* represents the known relationship between a drug and a side effect.

$$O={\left(O\right)}_{ij}\in R^{N_{r}\times N_{s}},$$ (3)

where *N*_*r*_ drugs have been associated with *N*_*s*_ side effects based on observed drug–side effect correlations. Each row is a drug, and each column is a side effect. (*O*)_*ij*_ is set to 1 if the drug *r*_*i*_ is associated with the side effect *s*_*j*_ and 0 otherwise.

To integrate the multiple associations between drug side effects, we constructed two heterogeneous graphs and denoted their adjacency matrices as *A* ^*chem*^ and *A* ^*dise*^. We built edges based on instances of known drug–side effect correlations, connecting *N*_*r*_ drugs and *N*_*s*_ side effect nodes based on the cases of known drug–side effect relationships. When (*O*)_*ij*_ = 1, we connect *r*_*i*_ to *s*_*j*_.

$$A^{k}=\left[\begin{array}{c} M^{k}O\\ O^{T}S \end{array}\right]\in R^{N_{v}\times N_{v}},$$ (4)

where *N*_*v*_ denotes the total number of nodes for drugs and side effects *N*_*v*_ = *N*_*r*_ + *N*_*s*_. The transposed matrix of *O* is defined as *O* ^*T*^. The similarities and associations associated with a drug or side effect node can be considered its attributes. Therefore, it can be considered an attribute matrix, denoted *H* ^*k*^.

### 2.3 Network-level enhanced topological representation learning

We built a drug–side effect association prediction model with an SCC and GAN with a representation-level attention (RLA) method. Modules based on GAN and SCC are used to learn the topological representation of network-level enhancements in drug–side effect heterogeneous graphs and the self-calibrated node–pair attribute representation, respectively.

#### 2.3.1 Enhanced topological representation learning based on GAN

Given two drug–side effects heterogeneous graphs, each network has its own unique characteristics, and we suggest an independent graph convolutional generation adversarial learning technique to individually encode the topological information of each heterogeneous graph. The module comprises the generator *G* and the discriminator *D* (Figure 1B). Adversarial learning between generators and discriminators forms a topological representation. Since the learning strategies are similar for drug–side effect heterogeneous graphs *G* ^*chem*^ and *G* ^*dise*^, we describe *G* ^*chem*^ as an example.

##### Generators based on graph convolutional selfencoders

We consider the attribute matrix $\overset{\wedge }{H}$ ^*chem*^ generated by the generator comprising all nodes as a false sample. The primary purpose of the generator is to make the generated matrices as close as possible to the original attribute matrix *H* ^*chem*^. As shown in Figure 1, the generator *G* encodes the provided attribute matrix.

##### Encoder

First, *A* ^*chem*^ is an adjacency matrix with node self-connections. ${\tilde{A}}^{\mathrm{chem}}$ can be obtained by Laplace normalization.

$${\tilde{A}}^{\mathrm{chem}}=\;{\left(D^{\mathrm{chem}}\right)}^{-\;\frac{1}{2}}A^{\mathrm{chem}}{\left(D^{\mathrm{chem}}\right)}^{-\;\frac{1}{2}},$$ (5)

where ${\left(D^{\mathrm{chem}}\right)}_{ii}=\sum_{j}{\left(A^{\mathrm{chem}}\right)}_{ij}\in R^{N_{v}\times N_{v}}$ and *D* ^*chem*^ is the degree matrix of *A* ^*chem*^. In order to learn the topological representation of network-level enhancements, the normalized adjacency matrix ${\tilde{A}}^{\mathrm{chem}}$ and the original attribute matrix *H* ^*chem*^ are fed together into the *L*-*th* coding layer of the generator, denoted as

$$H_{en\left(1\right)}^{\mathrm{chem}}=\varphi \left({\tilde{A}}^{\mathrm{chem}}H^{\mathrm{chem}}W_{en\left(1\right)}^{\mathrm{chem}}\right),$$ (6)

$$H_{en\left(L\right)}^{\mathrm{chem}}=\varphi \left({\tilde{A}}^{\mathrm{chem}}H_{en\left(L-1\right)}^{\mathrm{chem}}W_{en\left(L\right)}^{\mathrm{chem}}\right),$$ (7)

where *L* ∈ [2, *L*_*en*_], where *L*_*en*_ represents the overall number of coding layers, and *φ* represents the rectified linear unit (ReLU), the activation function. The weight matrices for the first and *L*-*th* layer graph convolution encoders are denoted $W_{en\left(1\right)}^{\mathrm{chem}}$ and $W_{en\left(L\right)}^{\mathrm{chem}}$ , respectively. In addition, $W_{en\left(1\right)}^{\mathrm{chem}}$ and $W_{en\left(L\right)}^{\mathrm{chem}}$ are the corresponding coded outputs for layers 1 and *L*, respectively. The output of the final coding layer is $H_{en\left(Len\right)}^{\mathrm{chem}}\in R^{N_{v}\times N_{f}}$ , where *N*_*f*_ is the dimension of the dimensionality of the reduced feature vector, which contains the representative information of all nodes, denoted $H_{en}^{\mathrm{chem}}$ .

##### Decoder

Decoder is a graph convolutional neural network-based framework for reconstructing the original matrix of drug side effect nodes. We mapped the topology representation back to the original space using a decoder. We then calculated the loss between the reconstructed matrix $\overset{\wedge }{H}$ ^*chem*^ and the original matrix *H* ^*chem*^ to obtain a better encoding for predicting drug–side effect associations. The decoding matrices $H_{de\left(1\right)}^{\mathrm{chem}}$ and $H_{de\left(L\right)}^{\mathrm{chem}}$ of the first and *L*-*th* layers are represented as follows:

$$H_{de\left(1\right)}^{\mathrm{chem}}=\varphi \left({\tilde{A}}^{\mathrm{chem}}H_{en}^{\mathrm{chem}}W_{de\left(1\right)}^{\mathrm{chem}}\right),$$ (8)

$$H_{de\left(L\right)}^{\mathrm{chem}}=\varphi \left({\tilde{A}}^{\mathrm{chem}}H_{de\left(L-1\right)}^{\mathrm{chem}}W_{de\left(L\right)}^{\mathrm{chem}}\right),$$ (9)

where $L\in \left[2,L_{de}\right]$ and *L*_*de*_ represent the overall quantity of the decoding layers. The weight matrices for the first and *L*-*th* decoding layers are denoted as $W_{de\left(1\right)}^{\mathrm{chem}}$ and $W_{de\left(L\right)}^{\mathrm{chem}}$ , respectively. $H_{de\left(1\right)}^{\mathrm{chem}}$ and $H_{de\left(L\right)}^{\mathrm{chem}}$ are the outputs of the corresponding decoding layers. The output of the final decoding layer $H_{de\left(L_{en}\right)}^{\mathrm{chem}}$ is renamed $\overset{\wedge }{H}$ ^*chem*^.

##### Discriminator based on MLP

The original matrix *H* ^*chem*^ and the reconstructed matrix $\overset{\wedge }{H}$ ^*chem*^ generated by the generator are provided as input to the discriminator *D* and are considered true and false samples, respectively. The discriminator attempts to distinguish between true and false samples, enabling the generator to obtain a more accurate topology representation of $H_{en}^{\mathrm{chem}}$ . The discriminator essentially evaluates the likelihood that the input sample is true or false. The discriminator should assign a high score to true samples and a low score to false samples. Let $D_{\left(L\right)}^{\mathrm{chem}}$ represent the discriminator’s hidden layer output. The input is flattened to obtain a vector *h* ^*chem*^ to feed into the discriminator to obtain the score distribution of the input samples.

$$D_{\left(1\right)}^{\mathrm{chem}}=\varphi \left(W_{D\left(1\right)}^{\mathrm{chem}}h^{\mathrm{chem}}+b_{\left(1\right)}^{\mathrm{chem}}\right),$$ (10)

$$D_{\left(L\right)}^{\mathrm{chem}}=\varphi \left(W_{D\left(L\right)}^{\mathrm{chem}}D_{\left(L-1\right)}^{\mathrm{chem}}+b_{\left(L\right)}^{\mathrm{chem}}\right),L=2,\ldots ,L_{D},$$ (11)

where *L*_D_ is the total number of hidden layers in the discriminator, $W_{D\left(L\right)}^{\mathrm{chem}}$ and $b_{\left(L\right)}^{\mathrm{chem}}$ are the layer’s weight matrix and bias vector, respectively, and $D_{\left(1\right)}^{\mathrm{chem}}$ and $D_{\left(L\right)}^{\mathrm{chem}}$ are the output of the corresponding hidden layer, respectively. The final layer’s activation function is *soft* max, while *φ* represents the ReLU activation function.

##### Optimization

The optimization goal of learning topological representation based on GANs is that the generator generates a reconstruction matrix as close to the original matrix as possible, the discriminator more accurately distinguishes the original matrix from the reconstruction matrix, and both form an adversarial relationship. Their optimization functions are as follows:

$$\begin{array}{c} \underset{G}{\min}\underset{D}{\max}V\left(D,G\right)=E_{H^{\mathrm{chem}}∼Pdata}\left[\log D\left(H^{\mathrm{chem}}\right)\right]\\ +E_{H_{en}^{\mathrm{chem}}∼Pdata}\left[\log\left(1-D\left(G\left(H_{en}^{\mathrm{chem}}\right)\right)\right)\right], \end{array}$$ (12)

where **E** represents the expectation and *P*_*data*_ represents the probability distribution of nodes in the original and reconstructed matrices. By maximizing the loss from the discriminator and minimizing the loss from the generator, they can achieve adversity with shared loss. The first expectation $E_{H^{\mathrm{chem}}∼Pdata}\left[\log D\left(H^{\mathrm{chem}}\right)\right]$ represents when the input is a true sample, and the second $E_{H_{en}^{\mathrm{chem}}∼Pdata}\left[\log\left(1-D\left(G\left(H_{en}^{\mathrm{chem}}\right)\right)\right)\right]$ represents when the input is a false sample generated by the generator. The algorithm *Adam* is used to improve the loss function. The two heterogeneous graphs are fed into separate GANs to learn the corresponding topological representation matrices $H_{en}^{\mathrm{chem}}$ and $H_{en}^{\mathrm{dise}}$ , respectively.

#### 2.3.2 Attention mechanism at the representation level

Given the topological representation matrices $H_{en}^{\mathrm{chem}}$ and $H_{en}^{\mathrm{dise}}$ of the nodes, the *i*-*th* row of $H_{en}^{\mathrm{chem}}$ ( $H_{en,i}^{\mathrm{chem}}$ ) is the topological vector of the node *v*_*i*_. Different aspects of $H_{en,i}^{\mathrm{chem}}$ contribute to the prediction of potentially important information. As the multiple topological representations have various contributions for the drug–side effect association prediction, we designed an attention at the representation level to obtain the informative representations. The attention scores of the *N*_*f*_ features of the node *v*_*i*_ form a score vector $s_{i}^{\mathrm{chem}}$ .

$$s_{i}^{\mathrm{chem}}=LeakyReLU\left(W_{\mathrm{fea}}^{\mathrm{chem}}*H_{en,i}^{\mathrm{chem}}+b_{\mathrm{fea}}^{\mathrm{chem}}\right),$$ (13)

where $s_{i}^{\mathrm{chem}}=\left\{s_{i}{,}_{1},s_{i}{,}_{2},s_{i}{,}_{3},\ldots ,s_{i},N_{f}\right\}$ represents the importance of the different features, *Leaky*ReLU represents the activation function, and $W_{\mathrm{fea}}^{\mathrm{chem}}$ and $b_{\mathrm{fea}}^{\mathrm{chem}}$ represent the learnable weight matrix and bias vector, respectively. *α*_*ij*_ is the normalized attention score of the *j*-*th* feature in $H_{en,i}^{\mathrm{chem}}$ .

$$\alpha_{ij}=\frac{\exp\left(s_{ij}^{\mathrm{chem}}\right)}{\sum_{k}\exp\left(s_{ik}^{\mathrm{chem}}\right)}.$$ (14)

Similarly, each feature of the vector $H_{en,i}^{\mathrm{dise}}$ is assigned an attention weight to form $s_{i}^{\mathrm{dise}}$ , which is defined as

$$s_{i}^{\mathrm{dise}}=LeakyReLU\left(W_{\mathrm{fea}}^{\mathrm{dise}}*H_{en,i}^{\mathrm{dise}}+b_{\mathrm{fea}}^{\mathrm{dise}}\right),$$ (15)

where *β*_*ij*_ is the normalized attention weight of $s_{i}^{\mathrm{dise}}$ .

$$\beta_{ij}=\frac{\exp\left(s_{ij}^{\mathrm{dise}}\right)}{\sum_{k}\exp\left(s_{ik}^{\mathrm{dise}}\right)}.$$ (16)

Therefore, the feature vector *v*_*i*_ obtained by augmenting the node with the attention mechanism can be expressed as *h*_*i*_.

$$h_{i}=\left(\alpha_{i}⊗H_{en,i}^{\mathrm{chem}}+H_{en,i}^{\mathrm{chem}}\right)+\left(\beta_{i}⊗H_{en,i}^{\mathrm{dise}}+H_{en,i}^{\mathrm{dise}}\right),$$ (17)

where “⊗” represents the element-by-element product operator. We perform an attention fusion operation on the feature vectors of each node in $H_{en}^{\mathrm{chem}}$ and $H_{en}^{\mathrm{dise}}$ to generate an enhanced topological representation $H_{en}\in R^{\left(N_{r}+N_{s}\right)\times N_{f}}$ throughout the network. Obtaining the topological embedding of the pharmacological side effect nodes for *r*_*i*_-*s*_*j*_, we extract the vectors corresponding to the *r*_*i*_ and *s*_*j*_ in *H*_*en*_, which are termed *x*_1_ and *x*_2_, respectively. As shown in Figure 1D, *x*_1_ and *x*_2_ form an *r*_*i*_-*s*_*j*_ enhanced topological embedding by stacking them on top and bottom.

$$X_{\mathrm{topo}}=\left[\begin{array}{c} x_{1}\\ x_{2} \end{array}\right]\in R^{2\times N_{f}}.$$ (18)

We obtain the topological representation *Z*_*topo*_ of *r*_*i*_-*s*_*j*_ by convolving *X*_*topo*_ fed into the two convolution-pooling layers.

### 2.4 Pairwise attribute learning based on self-calibrated convolutional neural networks

#### 2.4.1 Embedding construction of a pair of drug and side effect nodes

Given the similarity of the two drugs, we propose a strategy to form an embedding of the nodes’ attributes. The embedding process is depicted in Figure 1D using the example of *r*_*i*_ and *s*_*j*_. Given the matrices *M* ^*chem*^, *S*, and *O*, we first splice the *i*-*th* row ${\left(M^{\mathrm{chem}}\right)}_{i}$ of *M* ^*chem*^ and the *i*-*th* row ${\left(O\right)}_{i}$ of *O* to form the attribute vector $x_{1}^{\mathrm{chem}}$ , which is denoted as

$$x_{1}^{\mathrm{chem}}=\left[{\left(M^{\mathrm{chem}}\right)}_{i}\|{\left(O\right)}_{i}\right],x_{1}^{\mathrm{chem}}\in R^{N_{r}+N_{s}},$$ (19)

where ${\left(M^{\mathrm{chem}}\right)}_{i}$ represents how similar a drug’s chemical structure is to all others. ${\left(O\right)}_{i}$ provides details on how each adverse effect is related to the drug *r*_*i*_. “‖” is a splicing operation.

Then, the *j*-*th* row ${\left(O^{T}\right)}_{j}$ of *O* ^*T*^ and the *j*-*th* row ${\left(S\right)}_{j}$ of *S* are spliced to form the attribute vector *x*_2_, which is denoted as

$$x_{2}=\left[{\left(O^{T}\right)}_{j}\|{\left(S\right)}_{j}\right],x_{2}\in R^{N_{r}+N_{s}},$$ (20)

where ${\left(O^{T}\right)}_{j}$ and ${\left(S\right)}_{j}$ represent the relationship between *s*_*j*_ and all drugs and the similarity between *s*_*j*_ and all side effects, respectively. Finally, we stack $x_{1}^{\mathrm{chem}}$ and *x*_2_ to obtain the embedding matrix *x*_*chem*_.

$$X_{\mathrm{chem}}=\left[\begin{array}{c} x_{1}^{\mathrm{chem}}\\ x_{2} \end{array}\right],X_{\mathrm{chem}}\in R^{2*\left(N_{r}+N_{s}\right)}.$$ (21)

Similarly, given a drug similarity matrix *M* ^*dise*^, a side effect similarity matrix *S*, and a drug–side effect association matrix *O*, a second *r*_*i*_-*s*_*j*_ pairwise attribute embedding matrix $X_{\mathrm{dise}}\in R^{2*\left(N_{r}+N_{s}\right)}$ is obtained using the same embedding strategy. Finally, *X*_*chem*_ and *X*_*dise*_ are stacked to form the attribute embedding matrix $X_{\mathrm{att}}\in R^{2*2*\left(N_{r}+N_{s}\right)}$ .

#### 2.4.2 Self-calibrated pairwise attribute learning

For a pair of drug and side effect nodes, such as the drug *r*_*i*_ and the side effect *s*_*j*_, each feature of the node pair has the context relationship with the features around it. To capture the context relationship, a self-calibrated convolution-based attribute learning module was constructed. The module obtained the attribute embedding in a small latent space by utilizing convolution with larger receptive fields, and then, the embedding was used to guide the pairwise attribute learning in the original features space.

*X*_*att*_ undergoes average pooling to form a low-dimensional embedding of node pairs *L*.

$$L=APool\left(X_{\mathrm{att}}\right).$$ (22)

The feature transformation of *L* uses convolution operations.

$$X_{\mathrm{att}}^{\prime }=B\left[\varphi \left(W_{L}*L+b_{L}\right)\right],$$ (23)

where *B*[⋅] is a bilinear interpolation operation that maps the convolved feature map from the latent space back to the original space, “*” represents the convolution process, and *φ* represents the activation function ReLU. *W*_*L*_ and *b*_*L*_ represent the weight matrix and deviation vector, respectively. The feature graph $X_{\mathrm{att}}^{\prime }$ obtained in the latent space is used to calibrate the feature embedding *X*_*att*_ in the original space, forming the calibration weight *Y*_*cal*_.

$$Y_{\mathrm{cal}}=\left(W_{\mathrm{cal}}*X_{\mathrm{att}}+b_{\mathrm{cal}}\right)⊗\sigma \left(X_{\mathrm{att}}⊕X_{\mathrm{att}}^{\prime }\right),$$ (24)

where *σ* is the activation function. ⊕ and ⊗ represent the element-by-element addition and multiplication operations, respectively. *Y*_*cal*_ passes through a convolution-pooling layer to deeply fuse the calibrated features to form *Y*_*att*_.

$$Y_{\mathrm{att}}=\sigma \left(W_{\mathrm{cal}}*Y_{\mathrm{cal}}+b_{\mathrm{cal}}\right).$$ (25)

The original feature embedding *X*_*att*_ is convolved to form the original feature graph, comprising the original feature information. *X*_*att*_ is not padded to preserve and learn its edge information, and *Y*_*ori*_ is obtained after two convolutional layers. Finally, *Y*_*att*_ and *Y*_*ori*_ are joined to form the calibrated *r*_*i*_ − *s*_*j*_ attribute embedding matrix *Z*_*att*_.

$$Z_{\mathrm{att}}=\left\{Y_{\mathrm{att}},Y_{\mathrm{ori}}\right\}.$$ (26)

### 2.5 Final fusion and loss function

The learned topological representation *Z*_*topo*_ is first flattened into a vector *z*_*topo*_ and fed into the fully connected layer and *soft* max layer to obtain the association probability distribution of the drug *r*_*i*_ and the side effect *s*_*j*_.

$$score_{\mathrm{topo}}=softmax\left(W_{\mathrm{topo}}z_{\mathrm{topo}}+b_{\mathrm{topo}}\right),$$ (27)

where *W*_*topo*_ and *b*_*topo*_ are the weight matrix and deviation vector, respectively, and *soft* max is the activation function. In $score_{\mathrm{topo}}=\left[{\left(score_{\mathrm{topo}}\right)}_{0},{\left(score_{\mathrm{topo}}\right)}_{1}\right]$ , ${\left(score_{\mathrm{topo}}\right)}_{1}$ and ${\left(score_{\mathrm{topo}}\right)}_{0}$ represent the presence and absence of probabilities for an association between *r*_*i*_ and *s*_*j*_, respectively. There is a loss of cross entropy between the true label of the drug–side effect association and the expected likelihood *score*_*topo*_, which is defined as

$$loss_{\mathrm{topo}}=-\overset{T}{\underset{i=1}{\sum }}\left[y_{\mathrm{label}}\times \log{\left(score_{\mathrm{topo}}\right)}_{1}+\left(1-y_{\mathrm{label}}\right)\times \log\left(1-{\left(score_{\mathrm{topo}}\right)}_{0}\right)\right],$$ (28)

where *T* is a collection of training samples and *y*_*label*_ is the actual association between the nodes. *y*_*label*_ equals 1 if *r*_*i*_ is known to be associated with *s*_*j*_ and 0 otherwise.

The self-calibrating pairwise property representation *Z*_*att*_ is flattened into a vector *z*_*att*_ and fed into the fully connected and *soft* max layers. This module’s prediction score *score*_*att*_ and the loss function *loss*_*att*_ are defined as follows:

$$score_{\mathrm{att}}=soft\max\left(W_{\mathrm{att}}Z_{\mathrm{att}}+b_{\mathrm{att}}\right),$$ (29)

$$loss_{\mathrm{att}}=-\overset{T}{\underset{i=1}{\sum }}\left[{y_{\mathrm{label}}\times \log\left(score_{\mathrm{att}}\right)}_{1}+\left(1-y_{\mathrm{label}}\right)\times \log\left(1-{\left(score_{\mathrm{att}}\right)}_{0}\right)\right],$$ (30)

where ${\left(score_{\mathrm{att}}\right)}_{1}$ and ${\left(score_{\mathrm{att}}\right)}_{0}$ represent the *r*_*i*_-*s*_*j*_ correlation and non-correlation probabilities, respectively. The *Adam* algorithm was used to optimize the loss *loss*_*topo*_ and *loss*_*att*_. Finally, we weighted *score*_*topo*_ and *score*_*att*_ and fused them to obtain the final correlation prediction score.

$$score=\lambda \times score_{\mathrm{topo}}+\left(1-\lambda \right)score_{\mathrm{att}},$$ (31)

where hyperparameter *λ*(*λ* ∈ [0, 1]) is used to moderate the extent to which *score*_*topo*_ and *score*_*att*_ contribute to the final score.

## 3 Experimental evaluations and discussion

### 3.1 Evaluation metrics and parameter settings

The prediction performance of our model and other comparator models was assessed through five-fold cross validation. If a drug was observed to associate with a side effect by the biological experiments, the drug–side effect node pair may be regarded as a positive sample. On the other hand, all the unobserved drug–side effect node pairs are the negative samples. The number of positive samples and that of negative samples are 80,164 and 2,887,772, respectively, and their ratio is about 1:36. Thus, there is serious class imbalance for the positive samples and the negative ones. Five subsets of positive samples—four used for training and one for testing—were created by randomly equalizing all positive example samples. The same number of negative samples as the positive samples was selected for training, with the remainder used for testing.

The measures used in the evaluation process included the area under the receiver operating characteristic (ROC) curve (AUC), the area under the precision-recall curve (AUPR), and the recall rate for the top *k* candidates. The AUC is widely used to assess the performance of prediction models. Since there are much fewer negative than positive samples and the distribution is imbalanced, AUPR is more informative than AUC and helps assess the model’s performance. We separately calculated the AUC and AUPR of each fold during cross validation, and the final findings were calculated using the five-fold cross validation’s average AUC and AUPR. Typically, biologists choose the best candidates for additional validation. Therefore, we calculated the recall of the top *k* candidates (*k* ∈ [30, 60, … , 240]); the higher the recall, the more positive samples the prediction model correctly identifies.

The filter size within all the convolutional operations and the window size are 2 × 2. The GCN encoder has two encoding layers, and their feature dimensions are 2,500 and 1,500, respectively. The feature dimensions of the two decoding layers in the GCN decoder are set to 2,500 and 4,900, respectively. The dimensions of two hidden layers in the discriminator are 2,500 and 1,200. The topology representation fusion module contains two convolutional layers which have 16 and 32 filters. In the self-calibrated convolutional module, for the small feature space, the two convolutional layers have 1 and 32 filters, respectively. In terms of original feature space, the numbers of filters are 16 and 32, respectively. GGSC was developed on the PyTorch framework, and the server has a Nvidia GeForce GTX 2080Ti graphic card with 11 GB graphic memory.

### 3.2 Comparison with other methods

Six cutting-edge approaches for predicting pharmacological side effects were compared with GGSC, graph convolutional network-based risk stratification (GCRS), SDPred, Galeaon’s method, random walk on a signed heterogeneous information network (RW-SHIN), Ding’s method, and feature-derived graph regularized matrix factorization (FGRMF). In the cross-validation process, GGSC uses the same training and test sets as all comparison methods to compare the results more convincingly.

**FIGURE 3:**
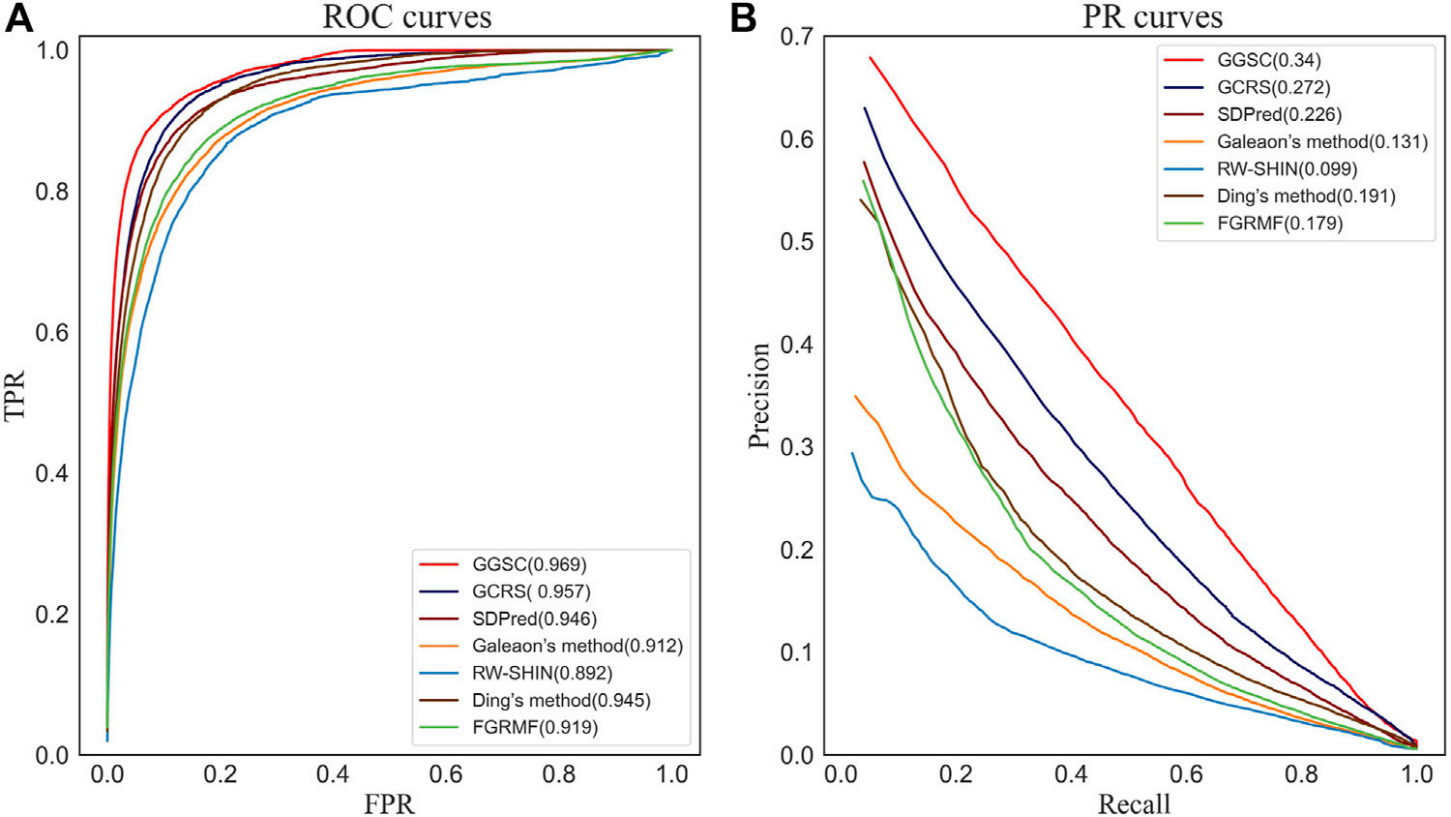
ROC curves and PR curves of our method and the compared methods for drug-side effect association prediction. **(A)** ROC curves **(B)** PR curves.

The average ROC and precision–recall (PR) curves for all methods using 708 drugs are shown in Figure 3. The average AUC of 0.969 of our GGSC model was 1.2% higher than that of the suboptimal GCRS, 2.3% higher than that of SDPred, 5.7% higher than Galeon’s method, 7.7% higher than RW-SHIN, 2.4% higher than Ding’s method, and 5.0% higher than FGRMF, respectively. Using 708 drugs, GGSC had the highest mean AUPR of 0.340, 6.8%, 11.4%, 20.9%, 24.1%, 14.9%, and 16.1% higher than GCRS, SDPred, and other methods, respectively.

After five-fold cross validation, we could obtain the average AUC and AUPR for each of the 708 drugs. We performed a Wilcoxon test on the 708 AUCs and AUPRs to determine whether performance differed significantly among methods (Table 1). These results showed that our method GGSC significantly outperformed the other prediction methods, when the *p*-value is always less than 0.05.

**TABLE 1:** Results of the paired Wilcoxon test on the AUCs and AUPRs over all the 708 drugs by comparing GGSC and other methods.

	GCRS	SDPred	Galeaon’s method	RW-SHIN	Ding’s method	FGRMF
*p*-value of AUC	1.7687-10	6.0316e-12	9.3621e-36	2.6478e-37	5.1475e-42	4.6142e-54
*p*-value of AUPR	6.2487e-14	6.3164e-15	3.2266e-32	7.3184e-42	8.2642e-46	7.3242e-58

**FIGURE 4:**
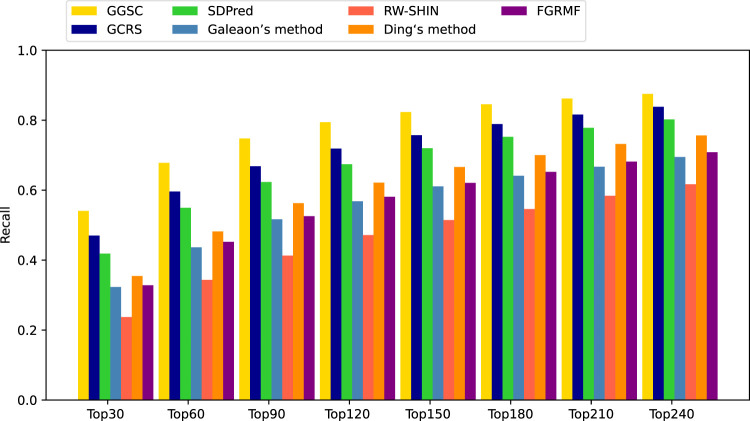
Recall rates of all the prediction methods at various top *k* values.

Among the compared methods, our GGSC method performed best, followed by GCRS. SDPred and Ding’s method integrate multiple drug similarities but ignore the heterogeneous graph’s topological information, so they do not perform as well as our GGSC method. FGRMF and Galeaon’s method are shallow prediction models that use matrix decomposition to predict drug-related side effects, which cannot effectively learn the deep associations between drug and side effect nodes, resulting in slightly worse performance. These findings show that RW-SHIN performs worse than other methods because it learns the topological information of medication nodes but not of side effect nodes. GGSC method’s better performance is mainly attributed to adversarial learning to obtain topological information and self-calibration learning to obtain node–pair properties. A higher recall of the top *k* candidate drug–side effect associations indicates that more true associations are correctly identified. Figure 4 shows that the GGSC method had consistently higher recall than the other methods for different values of *k*. When considering *k* = 30, GGSC had the highest recall (52.5%) and GCRS the second highest (47.0%).

Other methods had recall rates of 41.8%, 32.2%, 23.7%, 35.4%, and 32.8%, respectively. GGSC still performed best at values of *k* of 60, 90, and 120, with recall rates of 64.6%, 70.9%, and 75.2%, respectively. The second best performing method was GCRS, with recall rates of 59.6%, 66.8%, and 71.8%, respectively. The third best performing method was SDPred, with recall rates of 54.9%, 62.3%, and 67.4%, respectively. Ding’s method (48.1%, 56.2%, and 62.1%, respectively) and FGRMF (45.2%, 52.5%, and 58.1%, respectively) consistently outperformed Galeaon’s method (43.6%, 51.6%, and 56.8%, respectively). RW-SHIN consistently performed the worst, with recall rates of 34.3%, 41.2%, and 47.1%, respectively.

### 3.3 Ablation studies

We performed ablation experiments to confirm the contributions of the main innovations, including topological representation learning based on generative adversarial (TGA), RLA, and self-calibrated pairwise attribute (SCPA) learning (Table 2). The complete model, GGSC with TGA, RLA, and SCPA, performed best, with an AUC of 0.969 and AUPR of 0.340. For the model without TGA, the AUC and AUPR decreased by 1.5% and 3.8%, respectively, compared to the full model. These results show that topological representation learning helps to improve the model’s prediction performance. For the model without RLA, the AUC and AUPR fell by 1.0% and 4.2%, respectively, compared to the full model. The possible reason is that RLA assigns more weight to topological representations that are more informative, which helps the model capture more important features. For the model without SCPA, the AUC and AUPR declined by 2.6% and 3.1%, respectively, compared to the full prediction model. The main reason was that self-calibration enables learning more comprehensive information about the nodes’ neighboring nodes. This analysis demonstrates the respective contributions of TGA, RLA, and SCPA. The ablation experiment results show that SCPA learning provided the greatest enhancement to the drug–side effect association prediction model.

**TABLE 2:** Ablation study results of our method.

TGA	RLA	SCPA	Average AUC	Average AUPR
×	×	*✓*	0.954	0.302
*✓*	×	*✓*	0.959	0.298
*✓*	*✓*	×	0.943	0.309
*✓*	*✓*	*✓*	**0.969**	**0.340**

The bold value means the highest AUC (AUPR).

### 3.4 Case studies on five drugs and prediction of novel drug-related side effects

To further demonstrate our GGSC model’s ability to detect potentially relevant pharmacological adverse effects, we conducted case studies on five drugs: fluoxetine, lenalidomide, sumatriptan, risperidone, and aripiprazole. We obtained the drug’s associated candidate side effects and corresponding association scores, and all candidates were sorted in descending order. Tables 3–7 list the top 15 probable side effects for each of these five drugs.

**TABLE 3:** Top 15 candidate side effects of fluoxetine.

Drug	Rank	Side effect	Evidence
Fluoxetine	1	Anorexia	Drugcentral, MetaADEDB, Rxlist, SIDER
	2	Thrombocythemia	Rxlist
	3	Colonorrhagia	MetaADEDB, SIDER
	4	Cataract	Drugcentral, MetaADEDB, Rxlist, SIDER
	5	Hyponatremia	MetaADEDB, Rxlist
	6	Hypoventilation	MetaADEDB, Rxlist, SIDER
	7	Fibroids	MetaADEDB, Rxlist, SIDER
	8	Ecchymosis	MetaADEDB, Rxlist, SIDER
	9	Abdominal syndrome acute	Rxlist
	10	Neuritis	MetaADEDB, Rxlist, SIDER
	11	Optic neuritis	MetaADEDB, Rxlist, SIDER
	12	Purpura	MetaADEDB, Rxlist, SIDER
	13	Osteomyelitis	MetaADEDB, Rxlist, SIDER
	14	Hypertensive	Rxlist
	15	Somnolence	Drugcentral, MetaADEDB, Rxlist, SIDER

**TABLE 4:** Top 15 candidate side effects of lenalidomide.

Drug	Rank	Side effect	Evidence
Lenalidomide	1	Supraventricular arrhythmia	Rxlist
	2	Thrombophlebitis superficial	Rxlist, SIDER
	3	Dermatitis	MetaADEDB, Rxlist, SIDER
	4	Pemphigus	Drugcentral
	5	Abdominal pain	Drugcentral, MetaADEDB, Rxlist, SIDER
	6	Dysarthria	Drugcentral, MetaADEDB, Rxlist, SIDER
	7	Easy bruising	Rxlist
	8	Febrile neutropenia	Drugcentral, MetaADEDB, Rxlist, SIDER
	9	Abdominal tenderness	MetaADEDB, Rxlist, SIDER
	10	Stiffness	MetaADEDB, Rxlist, SIDER
	11	Allergic rhinitis	Drugcentral, Rxlist
	12	Angioedema	Drugcentral, MetaADEDB, Rxlist, SIDER
	13	Asthma	Drugcentral, MetaADEDB, Rxlist, SIDER
	14	Conjunctivitis	MetaADEDB, Rxlist, SIDER
	15	Dyspepsia	MetaADEDB, Rxlist, SIDER

**TABLE 5:** Top 15 candidate side effects of sumatriptan.

Drug	Rank	Side effect	Evidence
Sumatriptan	1	Abnormal pulse	Rxlist, SIDER
	2	Flatulence	MetaADEDB, Rxlist, SIDER
	3	Dehydration	MetaADEDB, Rxlist, SIDER
	4	Viral infection	Rxlist, SIDER
	5	Apathy	MetaADEDB, Rxlist, SIDER
	6	Keratitis	MetaADEDB, Rxlist, SIDER
	7	Sedation	Rxlist
	8	Chest pressure	MetaADEDB, Rxlist, SIDER
	9	Retinal vascular occlusion	MetaADEDB, SIDER
	10	Optic neuropathy	MetaADEDB, Rxlist
	11	Panic disorder	MetaADEDB, Rxlist, SIDER
	12	Enzymatic abnormality	MetaADEDB
	13	Discomfort	Drugcentral, MetaADEDB, Rxlist, SIDER
	14	Ulcer	MetaADEDB, Rxlist
	15	Convulsions	Rxlist

**TABLE 6:** Top 15 candidate side effects of risperidone.

Drug	Rank	Side effect	Evidence
Risperidone	1	Vomiting	Drugcentral, Rxlist, SIDER
	2	Transient blindness	Drugcentral, MetaADEDB
	3	Bradycardia	Drugcentral, MetaADEDB, Rxlist, SIDER
	4	Apnea	Rxlist
	5	Nightmares	Rxlist
	6	Mediastinal disorders	Drugcentral, MetaADEDB, Rxlist, SIDER
	7	Superficial phlebitis	Rxlist
	8	Hyperglycemia	MetaADEDB, Rxlist
	9	Rectal hemorrhage	Rxlist
	10	Female breast pain	MetaADEDB, Rxlist
	11	Flu	Rxlist
	12	Cyst	Drugcentral, MetaADEDB, Rxlist, SIDER
	13	Supraventricular extrasystoles	Rxlist, SIDER
	14	Generalized edema	Rxlist
	15	Dysphagia	Drugcentral, Rxlist, SIDER

**TABLE 7:** Top 15 candidate side effects of aripiprazole.

Drug	Rank	Side effect	Evidence
Aripiprazole	1	Dry eyes	Rxlist
	2	Decreased appetite	Drugcentral, Rxlist, SIDER
	3	Inguinal hernia	SIDER
	4	Anger	Drugcentral, MetaADEDB, SIDER
	5	Neuroleptic malignant	Drugcentral, MetaADEDB, Rxlist, SIDER
	6	Reflux	Rxlist, SIDER
	7	Thrombocythemia	MetaADEDB
	8	Dysphemia	Drugcentral, MetaADEDB, SIDER
	9	Groin pain	Drugcentral, MetaADEDB, Rxlist
	10	Hypoglycemic reaction	MetaADEDB, Drugcentral, Rxlist
	11	Conjunctivitis	MetaADEDB, Rxlist, SIDER
	12	Formication	MetaADEDB, SIDER
	13	Thrombophlebitis	MetaADEDB, Rxlist, SIDER
	14	Weight fluctuation	Drugcentral, MetaADEDB, Rxlist
	15	Abnormal dreams	MetaADEDB, Rxlist

Online database MetaADEDB containing comprehensive information on adverse drug events (ADEs), covering 744,709 associations between 8,498 drugs and 13,193 ADEs confirmed by clinical trials (Kuhn et al., 2016; Luo et al., 2017; Xuan et al., 2022). DrugCentral contains 4,927 drugs approved by regulatory authorities such as the European Medicines Agency, providing a resource for information on ADEs, indications, and more (Nair and Hinton, 2010; Wang et al., 2010; Davis et al., 2021). RxList contains information on drug descriptions and side effects in physicians’ articles and authoritative websites and supports > 5,000 drugs online (Hajian-Tilaki, 2013; Saito and Rehmsmeier, 2015). SIDER contains information on 1,430 drugs that have been marketed and their recorded ADEs from public documents and package inserts (Steigerwalt, 2015; Avram et al., 2021; Yu et al., 2021). Tables 3–7 show that 45 candidate side effects were recorded in MetaADEDB, 21 in DrugCentral, 60 in RxList, and 50 in SIDER. This result suggests that the drug candidates are associated with the corresponding side effects. The five drug case studies demonstrate GGSC’s ability to identify drugs’ potentially relevant side effects.

Following a thorough evaluation of the GGSC model’s performance, we used the training model to forecast 708 potential drug-associated side effects. Supplementary Table ST1 lists the top 30 potential side effects for each drug predicted by our model to aid biologists in their ongoing efforts to identify new side effects for drugs through biological testing.

## 4 Conclusion

We proposed a method to encode and fuse multiple types of similarities and associations from multiple heterogeneous graphs to predict drug-related candidate side effects. The constructed two drug–side effect heterogeneous graphs facilitate the formation of their specific topological embeddings based on the generative and adversarial strategy. The generator and the discriminator were constructed based on graph convolutional autoencoder and MLP, and then, the enhanced topological representations of the drug and side effect nodes were learned. The representation level attention was designed to assign higher weights to those more important topological representations. In the constructed self-calibrated convolutional neural network module, the pairwise features extracted from the small latent feature space are able to guide the feature learning in the original feature space. The cross-validation experimental results indicated that GGSC outperformed the compared prediction models in terms of both AUC and AUPR. Additionally, GGSC retrieved more realistic drug–side effect associations in the top-ranked candidate list, which makes it be more attractive to the biologists. GGSC’s ability in discovering the potential drug–side effect association candidates was further shown through case studies on five drug-related candidates.

## Data Availability

The original contributions presented in the study are included in the article/Supplementary Material; further inquiries can be directed to the corresponding author.
